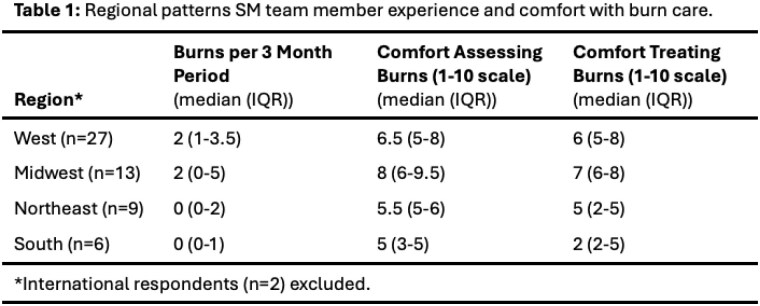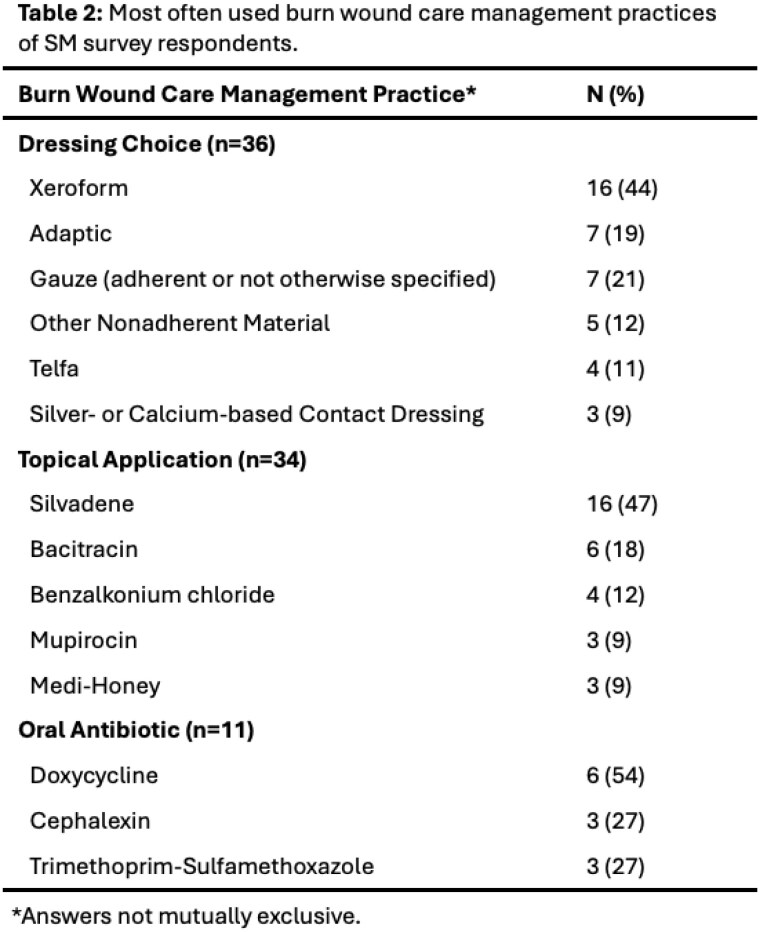# 120 Burn Care in the Street: The Current Landscape of Burn Care in Street Medicine

**DOI:** 10.1093/jbcr/iraf019.120

**Published:** 2025-04-01

**Authors:** Erin Ross, Alexis Coulourides Kogan, Maxwell Johnson, Haig Yenikomshian

**Affiliations:** University of California Keck School of Medicine; University of Southern California; University of California Keck School of Medicine; University of California Keck School of Medicine

## Abstract

**Introduction:**

People experiencing homelessness are at increased risk for serious burn injuries and face additional barriers to medical care. Street medicine (SM) programs, which provide direct medical care to unhoused people in shelters or the street, may be well positioned to bridge this gap in burn care. However, burn management is not featured in the SM literature, and SM clinician experience with burn wound management is not known.

**Methods:**

We surveyed SM team members to learn about their experiences with providing burn care in the street or shelter settings. Descriptive statistics were used to report findings from this cross-sectional survey.

**Results:**

60 survey respondents from 17 US states and two non-US countries included 18 physicians, 15 nurse practitioners or physician assistants, 15 registered nurses, 6 medical students, and 6 other client-facing team members. There was regional variability in frequency encountering burns in the street setting, and in comfort assessing and treating burns (Table 1). Burn wound management practices were also variable, though most clinicians treated burns with daily non-adherent dressings, and non-daily silver-based dressings were rare (Table 2). SM clinicians estimated prescribing oral antibiotics to a median 10% (IQR 0-38%) of burns survivors in the street. The most often reported barriers to burn care were prior negative experiences with emergency departments and transportation to burn centers.

**Conclusions:**

People experiencing homelessness are experiencing barriers in access to burn care. Although SM teams are already helping to deliver burn care to this population, experience and comfort with delivering burn care was variable.

**Applicability of Research to Practice:**

Formal partnerships between burn centers and SM teams through both burn care management education and integration of SM teams during the discharge process could help empower SM teams bridge the gap in burn care for people experiencing homelessness.

**Funding for the Study:**

N/A